# Multigene Profiling of Circulating Tumor Cells (CTCs) for Prognostic Assessment in Treatment-Naïve Metastatic Hormone-Sensitive Prostate Cancer (mHSPC)

**DOI:** 10.3390/ijms23010004

**Published:** 2021-12-21

**Authors:** Zachery R. Reichert, Tadas Kasputis, Srinivas Nallandhighal, Sophia M. Abusamra, Amy Kasputis, Saloni Haruray, Yugang Wang, Shamara Williams, Udit Singhal, Ajjai Alva, Frank C. Cackowski, Megan E. V. Caram, Phillip L. Palmbos, Sarah E. Yentz, David C. Smith, Joshi J. Alumkal, Todd M. Morgan

**Affiliations:** 1 Department of Internal Medicine, University of Michigan, Ann Arbor, MI 48109, USA; ajjai@med.umich.edu (A.A.); mveresh@med.umich.edu (M.E.V.C.); Ppalmbos@med.umich.edu (P.L.P.); syentz@med.umich.edu (S.E.Y.); dcsmith@med.umich.edu (D.C.S.); jalumkal@med.umich.edu (J.J.A.); 2 Department of Urology, University of Michigan, Ann Arbor, MI 48109, USA; tkasputi@med.umich.edu (T.K.); srnallan@med.umich.edu (S.N.); abusamra@med.umich.edu (S.M.A.); amgursky@med.umich.edu (A.K.); salonih@umich.edu (S.H.); wangyg03@gmail.com (Y.W.); shamawil@med.umich.edu (S.W.); usinghal@med.umich.edu (U.S.); 3 Department of Urology, Mayo Clinic, Rochester, MN 55901, USA; 4 Center of Excellence for Cancer Immunology and Immunotherapy, University of Michigan Rogel Cancer Center, Ann Arbor, MI 48109, USA; 5 Division of Medical Oncology, Karmanos Cancer Institute, Wayne State University, Detroit, MI 48201, USA; cackowskif@karmanos.org; 6 Lieutenant Colonel Charles S. Kettles VA Medical Center, Ann Arbor, MI 48105, USA

**Keywords:** prostate cancer, circulating tumor cells, CTCs, metastatic, hormone-sensitive, castration-sensitive, prognostic, gene expression

## Abstract

The substantial biological heterogeneity of metastatic prostate cancer has hindered the development of personalized therapeutic approaches. Therefore, it is difficult to predict the course of metastatic hormone-sensitive prostate cancer (mHSPC), with some men remaining on first-line androgen deprivation therapy (ADT) for several years while others progress more rapidly. Improving our ability to risk-stratify patients would allow for the optimization of systemic therapies and support the development of stratified prospective clinical trials focused on patients likely to have the greatest potential benefit. Here, we applied a liquid biopsy approach to identify clinically relevant, blood-based prognostic biomarkers in patients with mHSPC. Gene expression indicating the presence of CTCs was greater in CHAARTED high-volume (HV) patients (52% CTC_high_) than in low-volume (LV) patients (23% CTC_high_; * *p* = 0.03). HV disease (*p* = 0.005, q = 0.033) and CTC presence at baseline prior to treatment initiation (*p* = 0.008, q = 0.033) were found to be independently associated with the risk of nonresponse at 7 months. The pooled gene expression from CTCs of pre-ADT samples found AR, DSG2, KLK3, MDK, and PCA3 as genes predictive of nonresponse. These observations support the utility of liquid biomarker approaches to identify patients with poor initial response. This approach could facilitate more precise treatment intensification in the highest risk patients.

## 1. Introduction

One of the hallmarks of metastatic prostate cancer is its broad clinical heterogeneity, which can diminish the benefit of any given treatment strategy across an unselected patient population. For example, some men with metastatic hormone-sensitive prostate cancer (mHSPC) respond well to first-line androgen deprivation therapy (ADT) and remain in remission for many years. Other patients progress more rapidly within months. A prior prospective clinical trial in patients in mHSPC (GETUG-AFU15) [1] did not enrich for higher-risk disease and found that docetaxel was not beneficial. However, other studies enriched for more aggressive tumor subsets by recruiting a larger proportion of men with a higher volume of disease (CHAARTED) [2] or predominantly de novo metastatic disease (STAMPEDE, LATITUDE) [3,4]. Both of these studies demonstrated a clear benefit for docetaxel, leading to a change in the standard of care. These studies demonstrated the importance of understanding the biology and heterogeneity within a clinical cohort to design optimal clinical trials and, thus, outcomes for patients. The initial treatment decision and initiation at the time of mHSPC is important. Even though abiraterone has a clear survival benefit when initiated within 3 months of ADT starting per LATITUDE and STAMPEDE, when its start is delayed for 7 months and just given to those with a poor PSA response, the benefit is lost [4,5,6].

Clinical surrogates for biologic aggressiveness are imperfect. They may overestimate aggressiveness (e.g., 20% of high-volume (HV) patients per CHAARTED criteria do not progress on ADT monotherapy within 36 months) or do not address well men with prior local therapy, as only 3% of patients on STAMPEDE had prior local therapy and no patients on LATITUDE did [2,3,4,6]. There is a clear need for better prognostic biomarkers in patients with mHSPC to optimize the treatment of those with the highest-risk disease, while potentially sparing patients with less aggressive cancer from more toxic treatment regimens in the future. A tissue-based analysis was performed on mHSPC but utilized a mixture of prior prostate biopsies/prostatectomy specimens and metastatic tissue. This is due to the bone tropic nature of prostate cancer, which is less amenable to sequencing [7]. A nontissue method would be welcomed.

Approximately 1–10 circulating tumor cells/mL of blood are found in most men with metastatic prostate cancer and present a unique avenue for prognostic risk stratification in mHSPC [8,9,10,11,12,13,14,15]. Several studies in mHSPC have focused on circulating tumor cell (CTC) enumeration as a predictor for disease response (typically by PSA at 7 months) or duration of control (time to castration-resistant disease). These have ranged from 30 to 80 patients with various CTC thresholds (ranging from ≥2 or ≥5 cells/7.5 mL of whole blood). The largest study included 80 patients utilizing the CellSearch immunomagnetic bead capture system and subsequent imaging capture. Those with ≥5 cells developed castration resistant disease at a median of 17 months vs. those with <5 cells being 32 months (*p* = 0.007) [14]. All of these studies were performed prior to any intensification of upfront ADT within mHSPC, with agents such as docetaxel, abiraterone, apalutamide, or enzalutamide. Therefore, the impact of the presence of CTCs impact is unknown for current treatment paradigms. These studies did not analyze the CTCs further though. As circulating tumor cells (CTCs) recapitulate driver pathways in tissue samples, they may provide biologic insight [16].

We sought to use a pooled multiplex CTC gene assay to recreate the prognostic nature of CTC enumeration with a previously validated CTC probability score. We then sought to identify CTC-based genes associated with poor response to first-line therapy in the initial treatment of mHSPC. To do this, we established a prospective protocol (MiCoPilot) to collect peripheral blood samples prior to the start of any androgen deprivation therapy (ADT), enriched the samples for CTCs, and analyzed the gene expression of a preselected panel of androgen and prostate cancer-relevant genes. Response to primary metastatic therapy (either ADT alone or combined with other standard of care therapies) was assessed seven months after initiation of either a luteinizing hormone-releasing hormone (LHRH) agonist or antagonist. This timepoint was selected given the association of a poor PSA response (>4 ng/mL) at this endpoint with overall survival in prior studies of mHSPC [17,18]. We hypothesized that comparing patients who achieved a PSA < 4 ng/mL to those who did not respond to therapy within 7 months (by PSA combined with those who developed mCRPC or died within that time) and evaluating the impact of CTC measurements on other clinical features of aggressiveness would improve our ability to identify patients with aggressive mHSPC and obtain valuable biological insights.

## 2. Results

### 2.1. Patient Background

This study reported on the initial 58 patients with mHSPC enrolled in MiCoPilot (Table 1). The median age of patients was 73 years (interquartile range (IQR) = 11.8 years) and the median baseline PSA was 18 ng/mL (IQR = 72.8 ng/mL). There were 31 patients (53.4%) who did not have prior local treatment, and 27 (46.6%) who were treated with radical prostatectomy and/or prostate radiotherapy. LHRH agonist/antagonist monotherapy was used in 33 patients (56.9%). The other 25 patients (43.1%) received treatment intensification with chemotherapy or next-generation androgen-targeting agents within 3 months of ADT initiation. These consisted of abiraterone for 19 patients (5 HV and 14 CHAARTED low-volume (LV) patients), enzalutamide for 1 patient (HV), and docetaxel for 5 patients (all HV). Clinical and demographic characteristics are shown in Table 1, and clinical stratification by HV and LV groups showed no significant differences other than a trend toward more prior local treatment among patients with LV disease (*p* = 0.06).

### 2.2. Clinical Outcomes

A total of 43 patients (74.1%) were PSA responders, with fifteen patients (25.9%) being nonresponders at 7 months (Table 2). Nonresponders consisted of 13 patients with an incomplete PSA response (PSA > 4 at 7 months), of whom 4 developed mCRPC and 1 died all prior to 7 months. The remaining 2 nonresponders had a PSA response <4 at 7 months but had already developed mCRPC. Of the 15 nonresponders, 10 received ADT monotherapy and 5 received treatment intensification (abiraterone in one patient, enzalutamide in one patient, and docetaxel in three patients) (Table 2). Almost half of the HV patients (47.8%) were nonresponders, while 11.4% of the LV patients were nonresponders.

### 2.3. Detection of CTCs before Treatment Initiation Is Associated with Nonresponse Outcome at 7 Months following ADT Initiation

As described in the methods, as CTCs are not directly enumerated with this detection approach, CTC prevalence was approximated using five epithelial genes—EPCAM, DSG2, EGFR, KRT18, and KRT19—as per a previously established approach that provides an estimate of the probability that CTCs are present in the sample (termed CTC probability here and dichotomized as CTC_high_ and CTC_low_) [19]. Of the 58 patients analyzed in this study, 20 (34.5%) were categorized as CTC_high_ (Table 2). Half of the CTC_high_ patients were nonresponders. The prevalence of CTCs was found to be significantly greater in HV patients (52% CTC_high_) than in LV patients (23% CTC_high_; *p* = 0.03). Furthermore, CHAARTED HV status (*p* = 0.005) and CTC probability (high vs. low) before treatment initiation (*p* = 0.008) were associated with a nonresponse outcome at the 7 month endpoint using univariable logistic regression (Table 3). Using multivariable logistic regression (Table 3), HV disease and CTC probability were found to be independently associated with nonresponse at 7 months.

### 2.4. Prognostic Signature

#### 2.4.1. Unsupervised Hierarchical Clustering of all Patients Is Primarily Stratified by CTC Gene Expression

**Figure 1:**
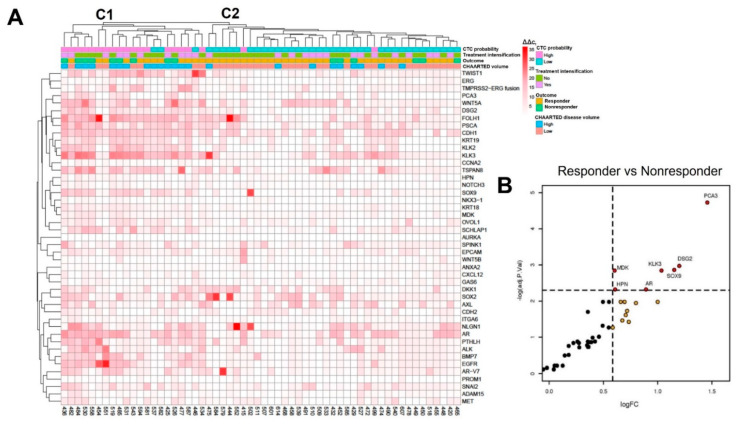
Heatmap of MiCoPilot patient gene expression prior to initiation of ADT (**A**). Unsupervised clustering of normalized gene expression was conducted using the ward.D2 clustering. CTC probability was determined using 5 epithelial genes as described in the methods. Patient characteristics, including treatment intensification and CHAARTED volume, and patient outcomes (responder/nonresponder) are annotated above the heatmap, and deidentified patient ID numbers and interrogated genes are annotated on the bottom and right of the heatmap, respectively. Volcano plot (**B**) showing differentially expressed genes that were significantly (FDR < 10% and LogFC > 0.585) enriched in nonresponders compared with responders.

Unsupervised hierarchical clustering of gene expression was conducted from multiplex qPCR data of CTC cDNA for the same 45 prostate cancer-related genes (Figure 1A). Gene expression was normalized per pooled CTC population by comparison to housekeeping genes. Heatmap annotations include CTC probability, treatment intensification, CHAARTED disease volume, and response outcome. The phylogenetic tree across the top of Figure 1 shows the stratification of the patient gene expression data primarily according to CTC probability, with 86% (18/21) of the patient samples on the left main branch (cluster 1—C1) categorized as CTC_high_ and 5% (2/38) of the patient samples on the right main branch (cluster 2—C2) categorized as CTC_high_. Furthermore, 43% (9/21) of the patients in C1 were nonresponders, while only 16% (6/37) of the patients in C2 were nonresponders.

Additionally, 67% (6/9) of the nonresponders in C1 were on ADT monotherapy, while 83% (5/6) of the nonresponders in C2 were on ADT. Volcano plots show differential gene expression results for responders vs. nonresponders (Figure 1B). HPN, MDK, KLK3, SOX9, AR, DSG2, and PCA3 were significantly upregulated in nonresponders (FDR < 10% and LFC > 0.585; Figure 1B). To further evaluate the association of individual genes with response outcomes at 7 months, receiver operator characteristic (ROC) analysis was conducted and AUC scores were calculated. Genes identified to be most conserved among all 58 patient samples included AR, DSG2, KLK3, MDK, PCA3, and SOX9 (Table 4).

#### 2.4.2. Highly Enriched CTC Populations Increase Sensitivity and Specificity of Predicting Disease Progression and Response to Therapy

**Figure 2:**
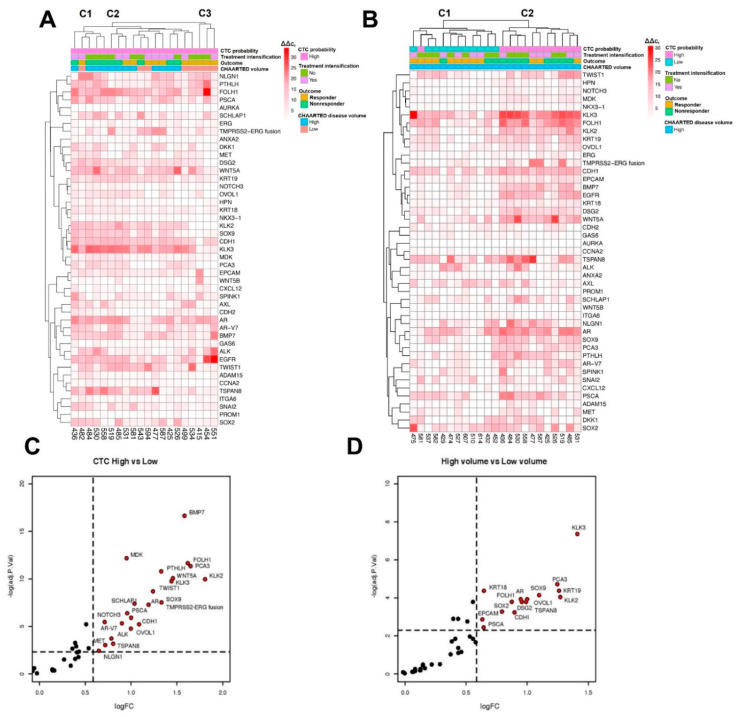
Unsupervised clustering of CTC_high_ patient (**A**) and HV patient (**B**) samples. Patient characteristics, including treatment intensification and CHAARTED volume, and patient outcomes (responder/nonresponder) are annotated above the heatmap, and deidentified patient ID numbers and interrogated genes are annotated on the bottom and right of the heatmap, respectively. Volcano plots showing differentially expressed genes based on CTC prevalence (**C**) and CHAARTED volume (**D**). Genes that passed FDR < 10% and LogFC > 0.585 cut-offs were determined significant and are highlighted on the plot.

We analyzed the cohort of CTC_high_ patient samples to assess patterns of gene expression in patients likely to have a substantial number of CTCs. Unsupervised hierarchical clustering of the CTC_high_ samples (Figure 2A) revealed three clusters. One (Figure 2A, C1) included five patients, of which four were nonresponders regardless of treatment intensification. The volcano plot comparing CTC_high_ versus CTC_low_ patients shows a number of different genes that were significantly upregulated in CTC_high_ patients (Figure 2C). *BMP7* appeared to be the most significantly upregulated gene in CTC high patients (FDR < 10% and LFC > 0.585) for this analysis (Figure 2C). Within the CTC_high_ cohort, *CDH1*, *FOLH1*, *HPN*, *KLK3*, *NKX3.1*, and *PCA3* (AUC scores: 0.90, 0.85, 0.81, 0.93, 0.85, and 0.85, respectively) were the best predictors of nonresponders (Table 4) and shared *KLK3* and *PCA3* in common with the total cohort analysis as top-scoring genes. The calculated accuracy of the combined prognostic six-gene set was 90% (sens: 0.89, spec: 0.91, PPV: 0.89, NPV: 0.91).

As a comparator, unsupervised clustering of the HV patient cohort revealed two distinct clusters (Figure 2B). These two clusters distinctly stratified between responders (C1, 75% responders) and nonresponders (C2, 73% nonresponders) and included those with and without intensification. The volcano plot showing the comparison between the HV and LV cohorts shows that 15 prostate cancer-related genes were significantly upregulated, and *KLK3* was the most significantly upregulated gene in patients with HV disease (FDR < 10% and LFC > 0.585) (Figure 2D). AUC analysis indicated that *AR*, *CDH1*, *DSG2*, *ITGA6*, *MDK*, and *PCA3* were found to be the six top-scoring genes in this cohort for prognosticating nonresponders (Table 4). The HV cohort shared several top-scoring genes with the total cohort (*AR*, *DSG2*, *MDK*, and *PCA3*) and with the CTC_high_ cohort (*CDH1* and *PCA3*).

#### 2.4.3. ADT Monotherapy

**Figure 3:**
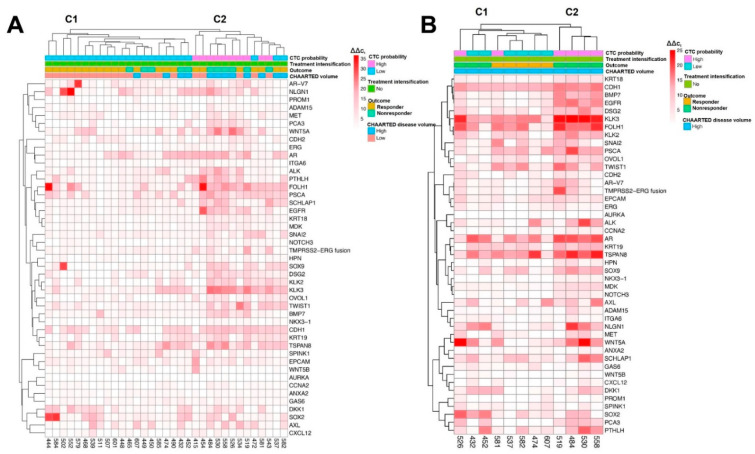
Unsupervised clustering of all patients (**A**) and HV patients (**B**) receiving ADT monotherapy. Patient characteristics, including treatment intensification and CHAARTED volume, and patient outcomes (responder/nonresponder) are annotated above the heatmap, and deidentified patient ID numbers and interrogated genes are annotated on the bottom and right of the heatmap, respectively.

Patients receiving ADT monotherapy were analyzed via unsupervised clustering independently in order to avoid treatment intensification drug effects (Figure 3A). The heatmap shows two distinct clusters, primarily segregated by CTC probability (100% of patients in C1 CTC_low_ and 77% of patients in C2 CTC_high_). Of the 20 patients in C1, 15 (75%) were responders, and 80% (16/20) of these were LV patients. Of the 5 nonresponders in C1, 2 were HV patients (40%), while of the 6 nonresponders in C2, 5 were HV patients (83%). In total, for ADT monotherapy, 83% (5/6) of CTC_high_ + HV patients were nonresponders versus 60% (6 of 10) for CTC_high_ and 58% (7 of 12) for HV patients. For CTC_low_ and LV on ADT monotherapy, 18% (3/17) of CTC_low_+LV patients were nonresponders versus 22% (5 of 23) for CTC_low_ and 19% (4 of 21) for LV patients. Among the patients receiving ADT monotherapy, *AR*, *EGFR*, *KLK3*, *MDK*, *PCA3*, and *SCHLAP1* were found to be the top six scoring genes for nonresponder prognostic ability. Gene expression signatures were also analyzed for mHSPC patients with HV disease who received ADT without any treatment intensification (Figure 3B). Interestingly, all the patients clustered in C2 were nonresponders (4/4), while only 38% (3/8) of patients in C1 were nonresponders. The degree of accuracy of the top six scoring genes for the HV, ADT monotherapy dataset in predicting nonresponders was markedly greater than those of the previously described sets (Table 4). *AR*, *HPN*, *KRT18*, *MDK*, *NLGN1*, and *PCA3* were the highest ranked genes in this set (AUC: 0.90, 0.87, 0.94, 1, 0.91, and 0.91, respectively) for nonresponder prognostic ability.

#### 2.4.4. Treatment Intensification

**Figure 4:**
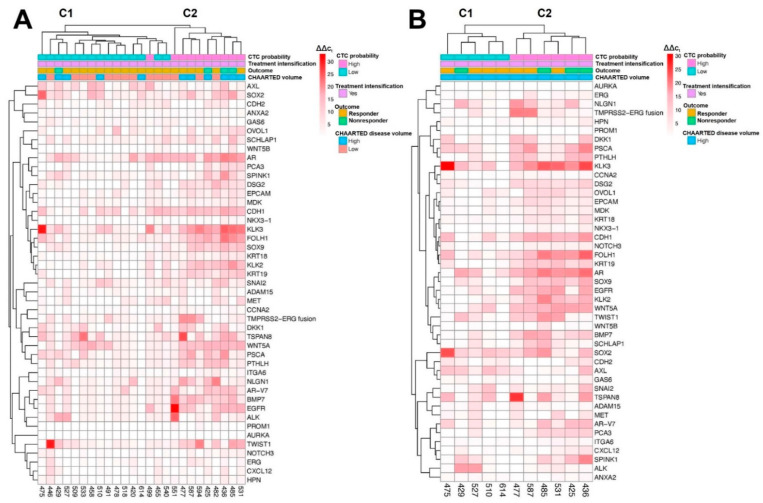
Unsupervised clustering of all patients (**A**) and HV patients (**B**) receiving treatment intensification within 3 months of initiating ADT. Patient characteristics, including treatment intensification and CHAARTED volume, and patient outcomes (responder/nonresponder) are annotated above the heatmap, and deidentified patient ID numbers and interrogated genes are annotated on the bottom and right of the heatmap, respectively.

We performed a similar analysis with the patients who received treatment intensification to analyze whether the gene set identifies cases with a higher risk of nonresponse in intensified patients, consistent with current treatment patterns. Of all 25 patients who received treatment intensification, 4 patients were nonresponders (all 4 patients were HV) at the 7 month endpoint. The heatmap for the intensified cohort of patients (Figure 4A) shows clustering of patients by CTC probability (100% CTC_high_ in C2 compared with 6% CTC_high_ in C1) and by disease volume (31% HV in C1 compared with 66% HV in C2). For CTC_high_ and HV with intensification, 50% (3/6) of CTC_high_ + HV patients were nonresponders versus 30% (3 of 10) for CTC_high_ and 36% (4 of 11) for HV. For CTC_low_ or LV with treatment intensification, no patients were nonresponders (0 of 10) of CTC_low_ /LV versus 7% (1 of 15) for CTC_low_ and 0% (0 of 14) for LV alone. Among the top scoring genes in this dataset, *AR* (AUC: 0.93) and *KLK3* (AUC: 0.84) were shared top genes between the intensified and ADT monotherapy datasets, while *AR*, *DSG2* (AUC: 0.79), *KLK3*, and *SOX9* (AUC: 0.87) were shared top genes between the intensified and total cohort datasets. Additional top scoring genes for this dataset included *AURKA* and *SPINK1* (Table 4). For HV patients who received intensification (Figure 4B), 75% of the nonresponders (3/4) clustered together in C2, with 50% of the patients in C2 being nonresponders. Top scoring genes within this dataset included AR (AUC: 0.93, shared top gene with all cohorts except CTC_high_), *CXCL12* (AUC: 0.82), *GAS6* (AUC: 0.80), *ITGA6* (AUC: 0.89, shared with HV cohort), *SPINK1* (AUC: 1.00, shared with intensification cohort), and *WNT5B* (AUC: 0.80) (Table 4).

## 3. Discussion

Risk stratification of an mHSPC patient currently leans on clinical characteristics (prior local therapy, disease volume, standard laboratory tests). For ADT therapy alone, our CTC_high_ mirrors the prognostic importance of CTC enumeration (cell/7.5 mL whole blood) as previously reported. This is the first observation that CTC volume retains prognostic importance for men receiving newer therapeutic agents (abiraterone and docetaxel predominantly) combined with ADT. In addition, the benefit seen with intensification was maintained between CHAARTED (HV vs. LN) and CTC (high vs. low)-designated men. Importantly, on multivariable analysis, CTCs appear to capture different information than CHAARTED radiographic disease volume (which is currently the most common method for prognosis). This may be attributed to unmeasured/unobserved disease volume as CTCs can capture nodal disease (which is not annotated in CHAARTED) or disease that is microscopic/invisible on standard imaging techniques. Otherwise, CTCs may have independent relevance as a reflection of biological processes that correspond with aggressive disease, such as the likelihood for further metastatic spread.

The biologic relevance of these CTCs was explored by pooled gene expression analysis. As the gene signal was standardized for housekeeping genes, the values reflect the population of CTCs on a cell for cell basis. *AR*, *DSG2*, *KLK3*, *MDK*, and *PCA3* were all genes of interest in this mHSPC cohort. The discovery of androgen signaling pathways was expected and *AR*, *KLK3*, and *PCA3* were prognostic. Absolute *AR* (androgen receptor) expression was prognostic, but interestingly, its splice variant (*AR-V7*) was not. This could be due to sample size and the rarity of *AR-V7* in hormone-naïve disease [20]. It could also be that these high-*AR* tumors in mHSPC have such a testosterone addiction that they will develop alternate signaling or steroid biosynthesis pathways more readily. Higher amounts of *KLK3* expression (kallikrein-related peptidase 3, also known as PSA) portend a worse response to abiraterone in mCRPC, and our data capture that in mHSPC [21]. *PCA3* (in blood or urine) has mainly been studied as a predictor for localized disease aggressiveness and has shown a decline with ADT or docetaxel in the metastatic setting [22,23,24]. Whether *AR*-directed agents or chemotherapy remain the best choice for those with high *AR*, *KLK3*, and *PCA3* patients remains to be tested, but could move this work from prognostic to predictive.

*MDK* (midkine) is intriguing as it has shown a role in driving castration resistance and has been previously found in CTCs [25]. It also may play a unique role in prostate cancer stem cells and neuroendocrine differentiation of prostate cancer [26,27]. These preclinical and clinical observations support targeted studies of *MDK* inhibition in mHSPC, particularly in combination with docetaxel where it has preclinical synergy.

*DSG2* appears to be a novel predictor in the metastatic setting. Despite encoding for a cell–cell adhesion protein, it acts independent of e-cadherin [28], and *DSG2* is prognostic in both primary prostate cancer [29] as well as other malignancies [30,31]. *DSG2* interacts with other tumorigenic pathways (e.g., *EGFR*) and may uniquely play a role in hormone-sensitive prostate cancer as it has not been identified as prognostic in CRPC studies [32,33].

There are several limitations to this study that should be highlighted. Key limitations of this study include sample size and the need for external validation of the primary study findings. Additionally, while a previously validated 7 month PSA response was used, validation using longer-term oncologic endpoints may be necessary, especially with treatment intensification or LV/CTC_low_ disease. Cell selection is potentially biased due to EPCAM-positive cell selection, which omits the analysis of non-EPCAM-expressing CTCs. This study also used a preselected gene set based off previous work in mCRPC. A gene-agnostic strategy with either the pooled cohort could provide an orthogonal approach to discover more novel pathways.

These limitations notwithstanding, the presented data highlight both disease volume (circulating and radiographic) and disease biology (through expression of relevant prostate cancer-related genes) as characteristics to potentially improve risk stratification for men initiating treatment for mHSPC. Additional strengths of this study include its prospective nature, the ability to assess ADT with current intensification treatment patterns, and a CTC pooling strategy that may recapitulate broader disease characteristics than a biopsy or single-cell analysis. With validation, the integration of this approach into clinical trials could test novel strategies for biomarker-based treatment intensification and de-intensification within mHSPC.

## 4. Materials and Methods

### 4.1. Patients and Inclusion Criteria

Between 2017 and 2020, men presenting with metastatic disease on standard CT, MRI or technetium bone scan, and a hematocrit ≥38 were consented. Subjects were required to have biopsy-confirmed cancer of the prostate or an elevated PSA (>20 ng/mL) with metastatic distribution (e.g., bone or pelvic/abdominal lymph node enlargement) consistent with prostate cancer in view of the treating provider. To correlate the molecular data with oncologic risk factors, multiple clinical parameters were collected (demographics, germline testing, prior and current therapies, and age at diagnosis) along with laboratory and imaging findings. All patient data were stored in REDCap v. 10.6.8 (Vanderbilt University), a HIPAA-compliant protected database.

Patients were required to have no current/recent LHRH agonist or antagonist exposure. Prior LHRH agonist/antagonist exposure in the curative setting (e.g., with radiation) was allowed if it was given >1 year prior to enrollment. No prior intermittent ADT periods for either biochemical recurrent prostate cancer or metastatic prostate cancer were allowed. Recent first-generation anti-androgens were allowed (e.g., bicalutamide).

### 4.2. Blood Collection

Peripheral blood was collected by standard venipuncture into an EDTA blood tube (BD Vacutainer). Visit frequency was per treating provider, necessitating a window of analysis for the 7 month ± 1 week outcomes endpoint (defined herein as just 7 months) [17,18]. Nonresponders were defined as patients who had a PSA > 4 ng/mL at the end of 7 months, developed mCRPC (per Prostate Cancer Working Group 3 definition), or died from any cause prior to 7 months. Responders were defined as patients who did not meet the above criteria (patients who achieved a PSA ≤ 4 ng/mL at the 7 month endpoint without evidence of progression).

### 4.3. CTC Isolation and Library Preparation

For the CTC-based evaluation, 4–15 mL of collected blood was processed using an anti-EPCAM immunomagnetic microbead enrichment approach. Briefly, whole blood was incubated with anti-EpCAM microbeads (Dynabeads, Life Technologies) for 30 min and washed 5 times with phosphate-buffered saline (PBS). Lysis buffer (Dynabeads, Life Technologies) was then added, and the supernatant containing the cell lysate was collected for expression analysis. Oligo(dT)25 mRNA Dynabeads (Life Technologies) were used for mRNA isolation, and cDNA transcriptome libraries were prepared using the Superscript III One-Step RT-PCR system (Life Technologies). Following library preparation and cDNA preamplification using TaqMan pooled primers (Applied Biosystems) and the preamplification master mix (Applied Biosystems), multiplex qPCR was performed for a total of 48 prostate cancer-related genes, including *AR, AR-V7, PSMA, PSA*, and *cytokeratins (8, 18, 19)*, as well as actin and tubulin as internal controls. Quantitative real-time PCR was performed using SYBR Green Mastermix (Life Technologies) on an Applied Biosystems QuantStudio 12k Flex real-time PCR machine. Gene expression signatures associated with response or resistance to therapy were identified [19,34].

### 4.4. Multiplex qPCR Data Analysis

All data analysis was performed using R version 3.6.3 (R Core Team (2020). R: A language and environment for statistical computing. R Foundation for Statistical Computing, Vienna, Austria. URL: https://www.R-project.org/ (accessed on 18 March 2021)). Raw cycle threshold (Ct) values were normalized using three housekeeping genes *ACTB*, *GAPDH*, and *TUBB* along with a set of internal controls to obtain ∆∆Ct values. Circulating Tumor Cell (CTC) scores were derived using the weights of five genes *EPCAM*, *DSG2*, *EGFR*, *KRT18*, and *KRT19* [19]. Samples with a CTC probability score <(−10) were classified as CTC_high_ and the rest as CTC_low_. Detailed methods, statistical data, and experimental verification regarding the determination of the CTC probability score can be found in a previously published study [19]. Differential gene expression analysis of the normalized data was performed using the lmFit function in R package limma. Genes with a false discovery rate (FDR) less than 10% and Log_2_ fold-change (LFC) greater than 0.585 (1.5 folds in linear space) were considered significant for further analysis. Unsupervised hierarchical heatmaps were generated using the R package pheatmap. Genes were clustered by Pearson correlation, and samples were clustered by Ward’s minimum variance method.

### 4.5. Statistics

Continuous correlative data were reported using means and standard deviations or medians with percentiles. Categorical covariates were reported using counts and frequencies. Prognostic or predictive associations of correlative biomarkers with response were assessed using logistic models with response to therapy and clinical progression as the outcomes and the correlative covariates and the independent predictors. Gene expression data were analyzed by receiver operator characteristic (ROC) analysis to determine the area under the curve (AUC) for each gene, excluding housekeeping genes, in relation to the 7 month outcomes as prognostic factors.

## Figures and Tables

**Table 1:** Demographic and clinical characteristics of the MiCoPilot cohort.

Characteristic	Total Cohort	LV Patients	HV Patients	*p*
	(n = 58)	(n = 35)	(n = 23)	(LV vs. HV)
Median age in years (IQR)	73 (66–78)	73 (66–77)	75 (64–86)	0.32 ^a^
Race *				
White	82.5% (47/57)	85.7% (30/35)	77.3% (17/22)	0.49 ^b^
Nonwhite	17.5% (10/57)	14.3% (5/35)	22.7% (5/22)	
**Gleason**				
≤7	24.1% (14/58)	34.3% (12/35)	13% (3/23)	0.11 ^c^
8–10	27.6% (16/58)	28.6% (10/35)	21.7% (5/23)	
Unknown	48.3% (28/58)	37.1% (13/35)	60.9% (14/23)	
**Germline Mutation**				
Pathogenic Mutation	5.1% (3/58) **	8.6% (3/35)	0	0.56 ^c^
VUS	8.6% (5/58)	8.6% (3/35)	8.9% (2/23)	
No Mutation	43.1% (25/58)	41.7% (15/35)	43.5% (10/23)	
Not Tested	44.8% (26/58)	44.4% (15/35)	47.8% (11/23)	
FH of Prostate Cancer	36.2% (21/58)	37.1% (13/35)	34.8% (8/23)	0.85 ^c^
**Disease at time of mHSPC**				
Median PSA in ng/mL, (IQR)	18 (8.7–92.8)	11.1 (8.5–47.0)	69.3 (9.6–166.5)	0.15 ^a^
Visceral Metastases	10.3% (6/58)	0	26.1% (6/23)	
**Prior Local Treatment**				
None	53.4% (31/58)	42.9% (15/35)	69.6% (16/23)	0.06 ^b^
Prostatectomy and/or Prostate Radiotherapy	46.6% (27/58)	57.1% (20/35)	30.4% (7/23)	
**Treatment in mHSPC**				0.6 ^b^
ADT Monotherapy	56.9% (33/58)	60% (21/35)	52.2% (12/23)	
ADT + Abiraterone	32.8% (19/58)	40% (14/35)	21.7% (5/23)	
ADT + Enzalutamide	1.7% (1/58)	0	4.3% (1/23)	
ADT + Docetaxel	8.6% (5/58)	0	21.7% (5/23)	
Metastasis-Directed Therapy	8.6% (5/58)	11.4% (4/35)	4.3% (1/23)	

* 1 unreported; ** CHEK2, BRCA2, CDKN2A, ^a^ Student t-test; ^b^ Fisher’s exact test; ^c^ chi-square test., IQR: interquartile range, FH: family history

**Table 2:** Patient outcomes at 7 months following initiation of ADT.

	Responders		Nonresponders
	PSA < 0.2	PSA = 0.2–4.0	PSA > 4 or Progression
	(n = 18)	(n = 25)	(n = 15)
**Treatment**			
ADT Monotherapy	50% (9/18)	56% (14/25)	66.7% (10/15)
ADT + Oral Agent (Abiraterone or Enzalutamide)	44.4% (8/18)	40% (10/25)	13.3% (2/15)
ADT + Docetaxel	5.6% (1/18)	4% (1/25)	20% (3/15)
**CHAARTED Volume**			
High Volume	11.1% (2/18)	40% (10/25)	73.3% (11/15)
Low Volume	88.9% (16/18)	60% (15/25)	26.7% (4/15)
**CTC status**			
CTC_high_	22.2% (4/18)	24% (6/25)	60% (10/15)
CTC_low_	77.8% (14/18)	76% (19/25)	40% (5/15)

**Table 3:** Univariable and multivariable logistic regression model for disease nonresponse.

Characteristic	Univariable				Multivariable		
	OR	95% CI	*p*-Value	False Discovery Rate ^1^	OR	95% CI	*p*-Value
Age	1.03	0.97–1.10	0.33	0.65	-		
Baseline PSA	1.00	1.00–1.01	0.59	0.79	-		
Family History of Prostate Cancer	0.97	0.26–3.34	0.96	0.96	-		
Race	0.28	0.07–1.18	0.08	0.22	-		
Prior Local Treatment	0.82	0.24–2.75	0.75	0.86	-		
Intensification	0.67	0.18–2.26	0.52	0.79	-		
CTCs	5.4	1.54–21.0	0.008	0.033	3.9	1.01–16.1	0.05
CHAARTED High Volume	5.96	1.67–25.1	0.005	0.033	4.44	1.15–19.6	0.036

OR = Odds Ratio, CI = Confidence Interval; ^1^ False discovery rate correction for multiple testing.

**Table 4:** Area under the curve (AUC) values are given for differentially expressed genes between responders and nonresponders across all patients (Total Cohort), as well as within key subgroups. These subgroups included patients with a high CTC probability (CTC_high_), patients who received ADT monotherapy (ADT_mono_), patients who received treatment intensification (Intensificaiton), all CHAARTED high-volume patients (HV), CHAARTED HV patients who received ADT monotherapy (HV ADT_mono_), and CHAARTED HV patients who received treatment intensification (HV Intensification). The top 6 scoring genes for each cohort are highlighted in **bold-underline** font, and the top 5 scoring genes in common between all cohorts are highlighted in grey in the gene column.

Gene	Total Cohort	CTC_high_	ADT_mono_	Intensification	HV	HV ADT_mono_	HV Intensification
*AR*	**0.76**	0.75	**0.75**	**0.93**	**0.90**	**0.90**	**0.93**
*AURKA*	0.58	0.64	0.52	**0.82**	0.56	0.60	0.79
*CDH1*	0.71	**0.90**	0.74	0.71	**0.77**	0.86	0.66
*CXCL12*	0.62	0.53	0.53	0.74	0.69	0.63	**0.82**
*DSG2*	**0.76**	0.79	0.74	**0.79**	**0.78**	0.77	0.71
*EGFR*	0.66	0.51	**0.78**	0.54	0.68	0.83	0.57
*FOLH1*	0.70	**0.85**	0.55	0.75	0.76	0.76	0.71
*GAS6*	0.50	0.57	0.69	0.60	0.59	0.67	**0.80**
*HPN*	0.70	**0.81**	0.75	0.69	0.74	**0.87**	0.59
*ITGA6*	0.60	0.54	0.55	0.71	**0.82**	0.70	**0.89**
*KLK3*	**0.76**	**0.93**	**0.76**	**0.84**	0.74	0.77	0.70
*KRT18*	0.69	0.72	0.71	0.71	0.77	**0.94**	0.63
*MDK*	**0.73**	0.79	**0.78**	0.70	**0.81**	**1.00**	0.66
*NKX3.1*	0.61	**0.85**	0.55	0.77	0.72	0.71	0.71
*NLGN1*	0.64	0.64	0.64	0.59	0.77	**0.91**	0.64
*PCA3*	**0.73**	**0.85**	**0.76**	0.76	**0.83**	**0.91**	0.73
*SCHLAP1*	0.65	0.63	**0.76**	0.60	0.59	0.60	0.50
*SOX9*	**0.74**	0.77	0.73	**0.87**	0.73	0.70	0.79
*SPINK 1*	0.58	0.54	0.55	**1.00**	0.69	0.53	**1.00**
*WNT5B*	0.64	0.52	0.56	0.77	0.71	0.66	**0.80**

## Data Availability

The data presented in the study may be available on request from the corresponding author in concordance with use limitations contained within the study’s consent document.
